# Identification and Characterization of 15 Novel *GALC* Gene Mutations Causing Krabbe Disease

**DOI:** 10.1002/humu.21367

**Published:** 2010-12

**Authors:** Barbara Tappino, Roberta Biancheri, Matthew Mort, Stefano Regis, Fabio Corsolini, Andrea Rossi, Marina Stroppiano, Susanna Lualdi, Agata Fiumara, Bruno Bembi, Maja Di Rocco, David N Cooper, Mirella Filocamo

**Affiliations:** 1 S.S.D. Lab. Diagnosi Pre-Postnatale Malattie Metaboliche, IRCCS G. GasliniGenova, Italy; 2 U.O. Neuropsichiatria Infantile - IRCCS G. GasliniGenova, Italy; 3 Institute of Medical Genetics, School of Medicine, Cardiff UniversityHeath Park, Cardiff CF14 4XN, UK; 4 Servizio di Neuroradiologia Pediatrica - IRCCS G. GasliniGenova, Italy; 5 Centro Regionale “Errori Congeniti del Metabolismo”, Università di CataniaCatania, Italy; 6 Centro di Coordinamento Regionale Malattie Rare, AO Universitaria “Santa Maria della Misericordia”33100 Udine, Italy; 7 S.S. Malattie Rare, U.O. Pediatria II, IRCCS G. GasliniGenova, Italy

**Keywords:** *GALC* mutations, Krabbe disease, leukodystrophy, genotype-phenotype analysis, founder mutation

## Abstract

The characterization of the underlying *GALC* gene lesions was performed in 30 unrelated patients affected by Krabbe disease, an autosomal recessive leukodystrophy caused by the deficiency of lysosomal enzyme galactocerebrosidase. The *GALC* mutational spectrum comprised 33 distinct mutant (including 15 previously unreported) alleles. With the exception of 4 novel missense mutations that replaced evolutionarily highly conserved residues (p.P318R, p.G323R, p.I384T, p.Y490N), most of the newly described lesions altered mRNA processing. These included 7 frameshift mutations (c.61delG, c.408delA, c.521delA, c.1171_1175delCATTCinsA, c.1405_1407delCTCinsT, c.302_308dupAAATAGG, c.1819_1826dupGTTACAGG), 3 nonsense mutations (p.R69X, p.K88X, p.R127X) one of which (p.K88X) mediated the skipping of exon 2, and a splicing mutation (c.1489+1G>A) which induced the partial skipping of exon 13. In addition, 6 previously unreported *GALC* polymorphisms were identified. The functional significance of the novel *GALC* missense mutations and polymorphisms was investigated using the MutPred analysis tool. This study, reporting one of the largest genotype-phenotype analyses of the *GALC* gene so far performed in a European Krabbe disease cohort, revealed that the Italian *GALC* mutational profile differs significantly from other populations of European origin. This is due in part to a *GALC* missense substitution (p.G553R) that occurs at high frequency on a common founder haplotype background in patients originating from the Naples region. © 2010 Wiley-Liss, Inc.

## INTRODUCTION

Krabbe disease, also known as globoid-cell leukodystrophy (GLD; MIM# 245200), is an autosomal recessive disorder resulting from the deficiency of galactocerebrosidase (GALC; E.C. 3.2.1.46), a lysosomal enzyme involved in the catabolism of galactosylceramide, a cerebroside located mainly in the myelin sheath. GALC defects lead to the accumulation of a cytotoxic metabolite (galactosylsphingosine or psychosine) which results in the apoptosis of myelin-forming cells [Wenger et al., 2001].

The majority of patients with Krabbe disease (around 85%-90% of cases) have the infantile form of the condition, presenting with extreme irritability, spasticity and developmental delay before the age of six months, with death occurring before the age of two. The remaining 10-15% of patients have a late-onset form of the disease, the onset of symptoms occurring between the age of 6 months and the fifth decade, and with slower disease progression. In the infantile form of Krabbe disease, the child, who appears normal for the first few months post-partum, presents with frequent crying and irritability, feeding difficulties, arrest/regression of motor and mental development, as well as seizures in some cases. Severe motor and mental deterioration follows, leading to decerebration. Peripheral neuropathy is typically present. In the late-onset forms of the disease, individuals can be clinically normal until weakness, vision loss and intellectual regression become evident; however, adult onset patients may show spastic paraparesis as the only symptom. Disease severity is variable, even within families. Further differentiation between late-infantile (onset age, six months to three years, severe clinical course), juvenile (onset age, three to eight years; more gradual progression lasting for years), and the adult-onset forms, is commonly employed (http://www.genetests.org) [Wenger, 2008]. Nerve conduction velocity studies have been reported to be normal in some adults with an enzymatically confirmed diagnosis. Brain magnetic resonance imaging (MRI) usually reveals deep white matter abnormalities consistent with a demyelinating process that involves the brainstem and cerebellum. Computed Tomography (CT) scans may reveal hyperdensity indicative of calcification involving the cerebellum, thalami, caudate, corona radiata and brainstem.

**Figure 1:**
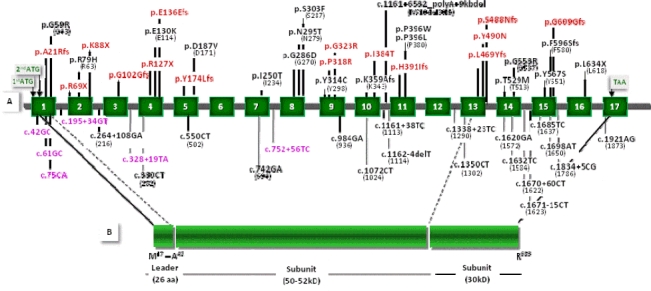
Distribution of the detected mutations and polymorphisms in relation to the *GALC* gene and GALC polypeptide. In the top half of the diagram (A), the map of the *GALC* gene depicts the positions of the seventeen exons (dark green boxes numbered 1 to 17) and associated unnumbered introns (gray lines). In the bottom half of the diagram (B), the schematic representation of the polypeptide shows the 26-amino acid signal (leader) sequence, and the two protein subunits (50-52 kDa and 30 kDa) predicted to be proteolytically processed from the precursor protein. All mutations encountered in this patient series are given in (A) above the gene schema, all polymorphisms below. Novel mutations and polymorphisms reported here are denoted in red and violet respectively. HGVS nomenclature guidelines stipulate amino acid numbering from the first methionine of the 42-residue signal sequence. Hence, HGVS nomenclature was used for the mutations and the polymorphisms reported here (with the traditional designations given in parentheses).

The galactosylceramidase gene *(GALC*; MIM# 606890], spanning 60 kb of genomic DNA on chromosome 14q31, comprises 17 exons and encodes a 3.8 kb mRNA (Figure 1). Two in-frame ATG translational start sites, located in exon 1 (Figure 1), give rise to distinct isoforms of GALC which are both translated. Although translational initiation at these alternative ATG codons yields protein precursors with 42-residue (NP_000144.2) or 26-residue leader sequences respectively, both precursors are processed to the 669-residue mature enzyme [Chen et al., 1993; Sakai et al., 1994; Luzi et al., 1995a]. The precursor protein, estimated to have a molecular mass of 80-kDa, is proteolytically processed into 30- and 50-kDa fragments in the extracellular medium. Neither the 50-nor the 30-kDa fragment, expressed *in vitro* in COS cells, have been demonstrated to possess GALC activity, an indication that the entire structure is probably necessary for enzymatic activity and that fragments expressed separately cannot subsequently associate to form an active enzyme [Chen et al., 1993; Chen and Wenger, 1993; Sakai et al., 1994, Nagano et al. 1998].

Although the *GALC* gene was identified more than 15 years ago, fewer than 80 mutations have been reported as a cause of Krabbe disease [http://www.hgmd.org; Stenson et al., 2009] and relatively few papers have addressed the issue of a genotype-phenotype relationship in this disorder. Clinical phenotypes can however differ quite markedly between individuals with the later-onset forms of the disease, including siblings harbouring the same *GALC* genotypes. The *GALC* mutational profile differs between European and Japanese Krabbe disease patients; thus, whilst the most common large deletion, c.1161+6532_polyA+9kbdel (IVS10del30kb) plus three other mutations [c.1586C>T, c.1700C>T, c.1472delA (1538C>T, 1652A>C, 1424delA)] together account for about 60% of alleles in patients of European ancestry with the classic infantile form [Kleijer et al., 1997; Wenger et al., 1997], these lesions are absent in their Japanese counterparts. By contrast, ∼30% of Japanese *GALC* alleles associated with the infantile form of the disease possess either c.683_694del12insCTC or c.2002A>C (635_646del12insCTC and 1954A>C) [Xu et al., 2006]. It should be noted that *GALC* mutations have traditionally been described on the basis of their amino acid position in the mature enzyme, with p.M17 being designated as the first residue. Current HGVS nomenclature recommendations however require proteins to be numbered from the first methionine of the complete 42-residue signal sequence. Hence, throughout this article, we ascribe HGVS designations to specific amino acid residues, with the traditional designations given in parentheses.

The median prevalence of Krabbe disease is estimated to be ∼1 in 100,000 births (1.00 × 10^−5^) with wide variations between countries: 1.35 in Netherlands, 1.21 in Portugal, 1.00 in Turkey, 0.71 in Australia, and 0.40 in the Czech Republic [Poupětová et al., 2010]. However, two quite separate inbred communities in Israel (the Druze and Moslem Arab populations) have an extremely high prevalence of the infantile form (about 1 in 100-150 live births), due to two non-identical mutations, c.1796>G and c.1630G>A (1748T>G and 1582G>A), respectively [Rafi et al., 1996]. The founder mutation c.169G>A (121G>A) is responsible for the other known Krabbe disease ‘hotspot’ in Catania (Sicily), this time associated with the late-onset form of the disease [Lissens et al., 2007].

In Italy, accurate epidemiological data for lysosomal disorders (LSDs) are not available owing to both the geographically wide dispersion of the patients between the various collaborating clinical/laboratory centres and the lack of any national register of rare diseases. However, the Gaslini laboratory has extensive experience in the analysis and diagnosis (both antenatal and postnatal) of LSDs, including Krabbe disease, going back over 35 years. Additionally, biobanked cell lines (“Cell line and DNA Biobank from patients affected by Genetic Diseases” at http://dppm.gaslini.org/biobank/) have enabled us to put together, between 1976 and 2009, unique collections of clinical samples and related data from a large series of families involving a total of 970 patients with 41 diverse LSD types/subtypes. Using the Biobank database, we have estimated the relative prevalence of Krabbe disease as ∼4% of the total number of LSD patients (data not shown). Although the examined time period and number of LSD types/subtypes differ slightly between published studies, our prevalence data are comparable to those reported from the Czech Republic and Australia (3.3% and 5%, respectively) [Poupetova et al., 2010; Meikle et al., 1999].

To date, the Krabbe disease collection within the Biobank includes 36 families. We have previously reported eight unrelated patients, in five of whom the second allele had remained undetermined [Selleri et al., 2000]. Here, we have characterized the underlying *GALC* gene lesions in a total of 30 unrelated Krabbe disease patients, in an analysis which included the complete genotyping of two patients from the previous study [Selleri et al., 2000]. This study therefore reports one of the largest mutational analyses of the *GALC* gene so far performed in a Caucasian population affected by Krabbe disease. It is apparent that the Italian mutational *GALC* profile differs significantly from other populations of European origin (http://www.genetests.org) [Wenger, 2008].

## MATERIALS AND METHODS

### Patients

The present series comprises a total of 30 unrelated patients with Krabbe disease. The diagnosis was suspected upon clinical evaluation and was supported by neuroimaging and neurophysiological findings. Most (27/30) patients underwent neuroradiological studies (brain MRI in 22/27 and CT scan in 13/27); nerve conduction velocity studies were performed in 14/30 patients. The characteristics of these patients and the main clinical findings are summarized in Table 1.

**Table 1:** Clinical, instrumental and molecular data encountered in 30 unrelated GLD patients

				Neurological findings		Neuroradiologic findings					
Pt no.	Sex	Age at onset	Symptoms at onset	Age at exam	Spastidty/ Other signs	MRI findings (WM changes)	CT scan findings (Calcification/WM changes)	NCVs	Sibling♣	GALC enzymatic activity § (% normal)	*GALC*Genotype**
1	F	1m	Failure to thrive	7m	+/ Truncal hypotonia	+	ND	Slowed		4.4	[c.1161+6532_polyA+9kbdel]+[?]

2	F	3m	Psychomotor regression	5m	+/ Truncal hypotonia	+ (Hypomyel)	ND	Slowed		8.2	[p.L469YfsX22]+[p.L469YfsX22]#

3	M	3m	Muscular hypertonia	6m	+/Nystagmus	+	+/ +	ND		0.3	[ p.G553R]+[p.G553R]#

4	M	3m	Irritability, muscular hypertonia	5m	+/Truncal hypotonia	+	ND	ND		14.7	[p.S303F]+[p.G553R]

5	M	3m	Muscular hypertonia. seizures	6m	+/ Truncal hypotonia	ND	+/ +	ND	yes	18.5	[p.E136EfsX35]+[p.E136EfsX35]

6	F	3m	Irritability, psychomotor regression	7m	+/ Truncal hypotonia	+	ND	Slowed		21.7	[p.K359AfsX3]+[p.Y567S

7	F	3m	Nystagmus, psychomotor regression	6m	+/Truncal hypotonia, poor tendon reflexes	+	ND	Slowed		3.2	[ p.Y174LfsX3]+[c.1161+6532_polyA+9kbdel]

8	M	4m	Irritability, muscular hypertonia	6m	+/Nystagmus	+	ND	Slowed	yes	0	[p.E130K]+[p.N295T]#

9	M	4m	Muscular hypertonia	6m	+/Truncal hypotonia, poor tendon reflexes	ND	+/ +	ND		11.1	[p.H391 IfsX65]+[p.H391 IfsX65]

10	M	4m	Muscular hypertonia	8m	+/ Truncal hypotonia	ND	-/ +	ND	yes	5	[p.T529M]+[c.1161+6532_ polyA+9kbdel]

11	F	4m	Muscular hypertonia	6m	+/ Truncal hypotonia	+ (Hypomyel)	ND	Slowed		8.4	[p.A21RfsX5]+[p.G553R]

12	F	4m	Irritability, muscular hypertonia	5m	+/ Truncal hypotonia	+ (Hypomyel)	+/ +	ND	yes	0	[p.Y314C]+[c.1161+6532_polyA+9kbdel]#

13	M	5m	Psychomotor regression	6m	+/Truncal hypotonia	+	ND	ND	yes	0	[ p.F596SfsX1 6] +[p.F596SfsX1 6]#

14	F	5m	Psychomotor regression, seizures	7m	+/ Truncal hypotonia	+ (Hypomyel)	ND	Slowed		0	[p.G553R]+[p.G553R]

15	F	5m	Irritability, muscular hypertonia	7m	+/ Truncal hypotonia	+ (Hypomyel)	+/ +	ND		0	[p.N295T]+[c.1161+6532_polyA+9kbdel]

16	F	5m	Psychomotor regression	7m	+/ Truncal hypotonia	ND	ND	ND		8.3	[p.R127X]+[p.G553R]##

17	F	5m	Muscular hypertonia	10m	+/ Truncal hypotonia	ND	ND	ND		4.8	[p.K88X]+[p.Y490N]#

18	M	5m	Psychomotor regression	9m	+/ Truncal hypotonia	+	+/ +	ND		8.8	[p.R396L]+[c.1161+6532_ polyA+9kbdel]

19	M	5m	Psychomotor regression	8m	+/ Truncal hypotonia	+	ND	ND	yes	18.2	[p.G553R]+[p.G553R]#

20	M	5m	Muscular hypertonia	7m	+/ Truncal hypotonia	+	ND	ND	yes	9.7	[p.L634X]+[c.1489+1G>A]

21	F	5m	Psychomotor regression	8m	+/ Truncal hypotonia	+	ND	ND	yes	0.71	[p.E130K]+[p.Y490N]

22	M	8m	Psychomotor regression	11m	+/ Truncal hypotonia	+	-/ +	ND	yes	13.2	[p.G102GfsX5]+[?]

23	M	8m	Psychomotor regression	11m	+/ Truncal hypotonia	ND	-/ +	ND	yes	0.26	[p.D187V]+[p.G323R]

24	F	10m	Psychomotor regression	12m	+/ Truncal hypotonia	+	-/ +	Slowed	yes	0	[p.G59R]+[?]#

25	M	11m	Irritability, muscular hypertonia	13m	+/ Truncal hypotonia	ND	-/ +	Slowed		10.1	[p.I250T]+[p.R396W]

26	F	3y6m	Gait disturbances and frequent falls	4y	+/Ataxia, poortendon reflexes	+	ND	Slowed		5	[ p.G286D]+[c. 1161 +6532_ polyA+9kbdel]#

27	F	3y6m	Gait disturbances and frequent falls	3y8m	+/Ataxia	+	-/ +	Normal		NA	[p.R69X]+[p.I384T]

28	M	4y	Gait disturbances and frequent falls	4y	+/Nystagmus	ND	ND	Slowed		13	[p.N295T]+[p.G609GfsX6]

29	F	4y	yReduced visual acuity	5y9m	+/-	+	-/ +	Normal	yes	0	[p.R79H]+[p.G553R]

30	M	26y	Gait disturbances and frequent falls	30y	+/-	+	ND	Slowed		5	[p.G286D]+[p.P318R]

Legend: Pt=Patient; m= month(s); y=year(s); #Genotype confirmation by parental DNAanalysis; ## only mother's DNAavailable; ND: Not Done; N=Normal; NA: Not Available **GenBank-EMBLaccession no. NM_000153.2 and no. NP_000144.2; Hypomyel= hypomyel inat ion; MRI= Magnetic resonance imaging, WM=white matter; CT= Computed tomography; NCVs= nerve conduction velocity study; ♣ indicates presence of other affected patients in the family; §Owingto the use of different assay met hodsand tissue samples, as reported intheMaterialsand Methods, enzyme act ivity valuesare expressed asa percentage of average control values.

A diagnosis of Krabbe disease was confirmed by enzymatic assay in homogenates of leukocytes or fibroblast cell lines using either tritium-labelled [H^3^] galactosylceramide (until 2004) or 6-hexadecanoylamino-4-methylumbelliferone-beta-D-galactoside (post-2004) as substrates. GALC activity values are provided for each patient in Table 1.

The patients in this series were diagnosed over a period of more than 30 years. The present study has been made possible thanks to the availability of the corresponding patient fibroblast/lymphoblast cell lines and/or DNA samples, cryopreserved within the laboratory “Cell Line and DNA Biobank from Patients affected by Genetic Diseases”. Following ethical guidelines, all samples obtained for analysis and storage required prior written informed consent using a form approved by the Local Ethics Committee.

### Cell culture

Fibroblast and lymphoblast cells were cultured according to standard procedures. The cell lines were cultured and maintained in RPMI medium (EuroClone, Gibco, Paisley, UK) containing 15% FCS and penicillin/streptomycin, in a humidified atmosphere containing 5% CO2 at 37°C.

### Molecular analysis

Genomic DNA was extracted using standard methods from cultured fibroblasts or lymphoblasts derived from the affected individuals and from peripheral blood leukocytes of the available family members. *GALC* gene exons and exon-intron boundaries were PCR amplified using specific primers designed by reference to the genomic sequence (GenBank-EMBL Accession No. NC_000014.8).

We initially screened for the large common Caucasian 30-kb *GALC* gene deletion (c.1161+6532_polyA+9kbdel) in all DNA samples, using three PCR primers in accordance with the previously reported method [Luzi et al., 1995b]. In the next step, a total of 16 PCR amplimers were tested on each patient, exons 2-3 being amplified simultaneously, as reported in Supp. Table S1. PCR amplification conditions were as follows: initial denaturation 2 min at 94°C, followed by 35 cycles amplification, denaturation at 94°C for 30 sec, annealing from 58°C to 62°C for 30 sec, and extension at 72°C for 40 sec using AmpliTaq DNA Polymerase (Applied Biosystems, Foster City, CA). Details of PCR-product sizes and annealing temperatures are reported in Supp. Table S1.

Total RNA was extracted from patient fibroblasts/lymphoblasts using an RNeasy mini kit (QIAGEN, Courtaboeuf, France) and reverse transcribed by means of an Advantage RT-for-PCR kit (BD Biosciences Clontech, Mountain View, CA, USA). RT-PCR was performed using sets of primers designed by reference to the GALC mRNA sequence (GenBank accession No. NM_000153.2). The RT-PCR set of primers employed is given in Supp. Table S1 together with the temperature profiles and expected product sizes. RT-PCR amplification conditions were as follows: initial denaturation 2 min at 98°C, followed by 30 cycles of amplification, denaturation at 98°C for 20 sec, annealing from 59°C to 65°C for 30 sec, and extension at 72°C for 30 sec, using Phusion High-Fidelity DNA Polymerase (Finnzymes, Keilaranta, Finland). These PCR products were cloned into the TOPO TA Cloning KIT (with pCR2.1-TOPO vector) (Invitrogen, San Diego, CA) according to the manufacturer's instructions.

Sequence analysis of PCR and RT-PCR products was performed in the forward and reverse directions using the ABI PRISM Big Dye Terminator Cycle Sequencing kit (Applied Biosystems, Foster City, CA). Sequences were analyzed on an ABI PRISM 3700 DNA Analyzer. Sequence alterations were confirmed by sequencing duplicate PCR products and/or by digesting PCR products with the specific restriction endonuclease whose recognition site had been concomitantly altered. If a given alteration had neither created nor destroyed a restriction site, PCR amplification was carried out by PCR-mediated site-directed mutagenesis that artificially introduced a new restriction enzyme cleavage site (Supp. Table S2 [Schwartz et al., 1991].

The issue of whether the novel *GALC* sequence alterations detected were causative mutations or neutral polymorphisms was addressed by (i) searching dbSNP (http://www.ncbi.nlm.nih.gov/SNP) for their presence, (ii) screening 100 alleles from healthy control subjects for each alteration, and (iii) employing the MutPred program [Li et al., 2009; Mort et al., 2010] (see below).

### *MutPred* analysis of *GALC* mutations and polymorphisms

The likely pathogenicity of missense mutations (and non-synonymous polymorphisms) identified in the human *GALC* gene was assessed by means of a computational model termed MutPred [Li et al., 2009; Mort et al., 2010]. MutPred was designed to model changes of structural and functional sites between wild-type and mutant protein sequences. Hence, MutPred can be used to generate hypotheses regarding the underlying molecular mechanism(s) responsible for disease pathogenesis

### Haplotype analysis

Segregation analysis of the identified polymorphisms was performed on all patients in the series including all patients coming from the same geographic area (Campania region).

### Mutation nomenclature

All mutations are described according to current mutation nomenclature guidelines (http://www.hgvs.org/mutnomen), ascribing the A of the first ATG translational initiation codon as nucleotide +1 [den Dunnen and Antonarakis, 2000; den Dunnen and Paalman, 2003]. Traditional amino acid residue numbering has nevertheless also been provided in parentheses.

## RESULTS AND DISCUSSION

A comprehensive clinical evaluation, facilitated by the results of a range of diagnostic procedures including neuroradiological, neurophysiological and enzymatic testing, was performed on 30 unrelated patients affected by Krabbe disease prior to characterization at the molecular genetic level. The clinical characteristics of these 30 patients and the main neuroradiological/ neurophysiological findings are summarized in Table 1. Apart from patient #2, of Moroccan origin, and patient #9, belonging to an itinerant Roma family, all patients were of Italian origin.

### Clinical aspects

As reported in Table 1, 21 patients (pts) were classified as having the infantile form of Krabbe disease (age at onset ranging from 1 to 5 months) while 9 were considered to have late-onset forms of the disease. In this latter group were 4 patients with the late-infantile form (age at onset ranging from 8 to 11 months), 4 patients with the juvenile form (age at onset ranging from 3 years 6 months to 4 years) and one adult-onset patient (onset at age 26 years). Except for the youngest patient who presented at 1 month of age simply with failure to thrive, muscular hypertonia (11/21) and psychomotor regression (9/21) variably associated with irritability (5/21) were the main presenting symptoms in the infantile-onset patients. Seizures and nystagmus were also observed at disease onset in two infantile patients (#5 and #7). Psychomotor regression was the presenting symptom in 3/4 patients with the late-infantile form, whereas irritability and muscular hypertonia occurred in the remaining patients from this group. Gait disturbances and frequent falls were the presenting symptoms in 4/5 patients with the juvenile (pts #26, #27, #28) and adult forms (pt #30) of the disease, whereas reduced visual acuity was the presenting symptom in the remaining case (pt #29).

Table 1 also reports the findings at neurological examination for all subjects, at variable age after disease onset, who invariably exhibited spasticity irrespective of the age at onset, variably associated with truncal hypotonia, nystagmus, ataxia and poor tendon reflexes. CT scans revealed white matter hypodensity in all 13 patients who underwent examination; this was associated with calcification in 6 patients (5/6 in the thalami, in one case in the periventricular white matter). Brain MRI revealed white matter changes in all studied patients which were consistent with hypomyelination in 5 cases (Table 1). Interestingly, this latter finding may represent a feature of lysosomal storage disorders with onset in the first months of life, when the process of myelination is particularly active, indicating that neuronal storage disorders may be primarily responsible for central nervous system hypomyelination [Di Rocco et al., 2005]. Nerve conduction velocity studies were slowed in 12/14 analyzed patients, but normal in 2 subjects with the juvenile form (pts #27 and #29) of the disease.

### *GALC* gene mutations in the Italian population

The *GALC* gene was investigated by sequencing analysis in all 30 unrelated Krabbe disease patients. As reported in Supp. Table S1, all 17 exons and most of the flanking intronic regions were analyzed. Additionally, the sequence analysis was extended to part of the 5′ untranslated region as well as of the untranslated region (Supp. Table S1). Table 1 summarizes the 27 distinct mutant genotypes encountered in this 30-patient series. Apart from 7 (23%) of the patients found to be homozygous for rare [p.G553R (G537R), p. F596SfsX16 (F580Sfs)] or novel [p.E136EfsX35, p.H391IfsX65, p.L469YfsX22] mutations, the remainder were rare compound heterozygotes, including 3 patients in whom the second mutant allele could not be identified. Among this latter group, 7 patients possessed the common large deletion of European origin (c.1161+6532_polyA+9kbdel) in *trans* to rare missense mutations p.G286D (G270D), p.N295T (N279T), p.Y314C (Y298C), p.R396L (R380L), p.T529M (T513M), a new frameshift (p.Y174LfsX3), or a still-unidentified mutation. As shown in Table 2, the *GALC* mutational profile was highly heterogeneous, being characterized by a total of 33 distinct mutant alleles including 15 previously unreported alleles; three mutant alleles still remain to be identified. Apart from 4 novel missense mutations (p.P318R, p.G323R, p.I384T, p.Y490N), 73% of the newly described mutations were expected to affect mRNA processing. These comprised 7 frameshift mutations resulting from 3 microdeletions (c.61delG, c.408delA, c.521delA), 2 indels (c.1171_1175delCATTCinsA, c.1405_1407delCTCinsT) and 2 microduplications (c.302_308dupAAATAGG, c.1819_1826dupGTTACAGG), respectively; 3 nonsense mutations (p.R69X, p.K88X, p.R127X); and a splicing mutation at the donor site of intron 13 (c.1489+1G>A) which induced the partial skipping of exon 13. Figure 1 depicts the location of the various mutations detected in relation to the *GALC* gene and its protein product. It is evident that the *GALC* mutations associated with severe clinical phenotypes are spread throughout the gene without any preferential clustering within the sequence encoding the 30-kD subunit of the mature GALC protein, thought to be critical for the synthesis of catalytically active enzyme [Rafi et al., 1995 and 1996].

**Table 2:** Characteristics of the *GALC* gene gene mutations identified in the 30 Krabbe disease patients and MutPred analysis of the missense mutations

					MutPred analysisof missense mutations#		
Location	Site of nucleotide substitution*	Amino acid change**	Traditional numbering nucleotide (amino acid) substitution	Type of mutation	Probability of deleterious mutation	Confident *in silico* hypotheses	References
Ex. 1	**c.61delG**	**p.A21RfsX51**		**Frameshift**	-		Present study
	
	c.175G>C	p.G59R	127G>C (G43R)	Missense	0.92	None	Fu et al.(1999)
	
	**c.205T>C**	**p.R69X**		**Nonsense**	-		Present study

Ex.2	c.236G>A	p.R79H	188G>A(R63H)	Missense	0.85	None	De Gasperi et al. (1996)

	**c.262A>T**	**p.K88X**		**Nonsense**	-		Present study

Ex.3	**c.302_308dupAAATAGG**	**p.G102GfsX5**		**Frameshift**	-		Present study

	**c.379C>T**	**p.R127X**		**Nonsense**	-		Present study
	
Ex. 4	c.388G>A	p.E130K	340G>A(E114K)	Missense	0.80	Gain of molecular recognition feature (MoRF) binding (P=0.0047), Gain of methylation at E130 (P=0.0114), Gain of ubiquitination at E130 (P=0.0269)	Lissens et al.(2007)

	**c.408delA**	**p.E136EfsX35**		**Frameshift**	-		Present study
	
Ex. 5	**c.521delA**	**p.Y174LfsX3**		**Frameshift**	-		Present study

	c.560A>T	p.D187V	512A>T(D171V)	Missense	0.71	None	Luzi et al. (1996)

Ex. 7	c.749T>C	p.I250T	701T>C(I234T)	Missense	0.79	Gain of protein disorder (P=0.0309), Loss of beta sheet secondary structure (P=0.0392)	De Gasperi et al. (1996)
	
	c.857G>A	p.G286D	809G>A(G270D)	Missense	0.98	None	Furuya et al. (1997)

Ex. 8	c.884A>C	p.N295T	836A>C(N279T)	Missense	0.80	None	Wenger et al. (1997)
	
	c.918C>T	p.S303F	870C>T(S287F)	Missense	0.83	None	Wenger et al. (1997)
	
	c.941A>G	p.Y314C	893A>G(Y298C)	Missense	0.89	None	De Gasperi et al. (1996)

Ex. 9	**c.953C>G**	**p.P318R**		**Missense**	0.79	Increased solvent accessibility (P=0.0179), Loop > Helix secondary structure change (P=0.0259)	Present study
	
	**c.967G>A**	**p.G323R**		**Missense**	0.91	None	Present study
	
Ex. 10	c.1075_1084delAAGACAGTTG	p.K359AfsX3	1027_1036delAAGACAGTTG§(K343AfsX3)	Frameshift	-		Wenger et al. (1997)
	
	**c.1151T>C**	**p.I384T**		**Missense**	0.74	None	Present study

Intr. 10	c.1161+6532_polyA+9Kbdel		IVS10del30kb	Deletion	-		Rafi et al. (1995)

	**d 171_1175delCATTCinsA**	**p.H391IfsX65**		**Frameshift**	-		Present study

Ex.11	c.1186C>T	p.R396W	1138C>T(R380W)	Missense	0.96	Loss of protein disorder (P=0.0371)	Wenger et al. (1997)
	
	c.1187G>T	p.R396L	1139G>T(R380L)	Missense	0.95	Gain of ubiquitination at K393 (P=0.0452)	Selleri et al. (2000)

Ex. 13	**c.1405_1407delCTCinsT**	**p.L469YfsX22**		**Frameshift**	-		Present study
	
	**c.1468T>A**	**p.Y490N**		**Missense**	0.68	Loss of phosphorylation at Y490 (P=0.0245)	Present study

Intr. 13	**c.1489+1G>A**	**p.S488NfsX200**		**Splicing**	-		Present study

Ex. 14	c.1586C>T	p.T529M	1538C>T(T513M)	Missense	0.68	None	Wenger et al. (1997)
	
	c.1657G>A	p.G553R	1609G>A(G537R)	Missense	0.91	Sheet > Helix secondary structure change (P=0.0151)	De Gasperi et al. (1999)

	c.1700A>C	p.Y567S	1652A>C(Y551S)	Missense	0.73	None	Wenger et al. (1997)
	
Ex. 15	c.1787delT	p.F596SfsX16	1739delT(F580SfsX16)	Frameshift	-		Selleri et al. (2000)
	
	**c.1819_1826dupGTTACAGG**	**p.G609GfsX6**		**Frameshift**	-		Present study

Ex. 16	c.1901delT	p.L634X	1853delT(L618X)	Nonsense	-		Wenger et al. (1997)

Legend: Ex=exon; Intr=intron; *GALC gene GenBank-EMBL accession no. NM_000153.2; **GALC gene GenBank-EMBL accession no. NP_000144.2; the novel mutations are given in bold; §mutation reported as c.1026del10 by Rafi et al., unpubished observations [Wenger et al., 2007]. #Li et al., (2009) and Mort et al., (2010).

### Relative frequencies of the *GALC* mutations and evidence for a founder effect involving the p.G553R (G537R) mutation

In agreement with previous studies on individuals of European ancestry (http://www.genetests.org) [Wenger, 2008], the large deletion (c.1161+6532_polyA+9kbdel) was confirmed to be the most frequent *GALC* mutation in the Italian population (taking together 6 previously reported deletion alleles [Selleri et al., 2000] with the 7 deletion alleles from the present series). However, while in other populations this large deletion has been shown to have a higher frequency, being respectively 52% of disease alleles in Dutch patients [Kleijer et al., 1997] and 44% of disease alleles in patients with Northern European ancestry [Luzi et al., 1995b; Rafi et al., 1995], in our series we found that this same deletion [c.1161+6532_polyA+9kbdel] barely accounts for 18% of disease alleles in our own series. Moreover, our findings contrast with the three other common mutations [p.T529M (T513M), p.Y567S (Y551S) and c.1472delA (1424delA)], expected to account for ∼15% of *GALC* alleles in the European population (http://www.genetests.org) [Wenger, 2008]; in our series, the two missense mutations were noted only once each (corresponding to 3.2% of alleles) whereas the microdeletion c.1472delA was not detected in our series at all. Instead, the second most frequent allele was p.G553R (G537R), previously only reported in one other family originating in southern Italy [De Gasperi et a1., 1999]. Since all seven patients harbouring p.G553R (G537R) (Table 1, pts #3, #4, #11, #14, #16, #19, #29) shared a common geographical origin around Naples (Campania region, southern Italy), we analysed their haplotype backgrounds. Among the various identified polymorphisms, it was evident that two very rare SNP alleles, namely rs74073730 [c.1072C>T (1024C>T)] and rs74076317 [c.1338+23T>C (1290+23T>C)], were exclusively present in all 7 p.G553R-bearing patients (Supp. Table S3). Both SNP alleles, originally identified by massively parallel sequencing [Bentley et al., 2008], are extremely rare; dbSNP gives their heterozygosity as 'not known' (http://www.ncbi.nlm.nih.gov/projects/SNP/snp_ref.cgi?rs=rs74073730). Hence the degree of linkage disequilibrium between the rare SNP alleles and the p.G553R disease allele could not be formally measured. However, we did exclude the presence of the rare SNP alleles in the remainder of the Krabbe disease patients, including three other individuals from the Campania region, who carried different *GALC* mutant genotypes (Table 1, pts #13, #22, #24.). It is therefore likely that the p.G553R (G537R) mutation occurred on the rs74073730(T) -rs74076317(C) haplotype background of a common founder ancestor. Identity-by-descent would account for the unexpectedly high number of an otherwise extremely rare *GALC* mutation in Italian Krabbe disease patients.

In addition to the putative pathological mutations, a total of 24 *GALC* polymorphisms were identified within either the exons, introns or 5′ untranslated region (5′ UTR). Novel SNPs were noted in the 5′ UTR (1), within the coding region (2) and in the introns (3) (Figure 1; Supp. Table S3). The allele frequencies of the new variants, derived from 200 normal Italian controls, are given in Supp. Table S3.

### *MutPred* analysis

In an attempt to establish the functional relevance or otherwise of the novel *GALC* missense mutations detected, we employed the *in silico* analysis tool, *MutPred* [available at http://mutdb.org/profile/; Li et al., 2009; Mort et al., 2010]. To assess the predictive power of this bioinformatic approach, the analysis was performed on both the newly identified missense mutations and the previously reported deleterious missense mutations. For the known pathological missense mutations listed in Table 2, *MutPred* successfully predicted all 18 (probability threshold >0.65) to be deleterious and generated confident *in silico* hypotheses for 39% (7 out of 18) of these mutations. Further investigation of the 4 novel missense mutations (p.P318R, p.G323R, p.I384T, p.Y490N) identified in the present study, indicated all four to have a high probability (>0.68) of being deleterious; *in silico* hypotheses for the underlying mechanism were generated for two of them. Thus, the novel missense mutation p.P318R is predicted to impact upon protein structure in terms of both solvent accessibility (P = 0.0179) and secondary structure (loop → helix; P = 0.0259) whereas the novel missense mutation p.Y490N is predicted to disrupt a phosphorylation site at this residue (P = 0.0245).

By contrast, none of the missense polymorphisms listed in Supp. Table S3, were predicted to be deleterious (probability threshold < 0.65). However, it should be noted that, according to *MutPred*, both the novel missense polymorphism p.A21P and the previously identified missense polymorphism [p.T641A (T625A)] have probabilities of being deleterious that lie close to the notional threshold (0.62 and 0.58 respectively) suggesting that they could be of functional significance. None of the missense polymorphisms were however predicted to disrupt any structural or functional sites in the GALC protein.

### Analysis of evolutionary conservation of amino acid residues affected by missense mutations

Additional support for the pathological/functional significance of the missense mutations (and potentially some of the polymorphisms) identified in the present study, came from the analysis of the extent of evolutionary conservation of the mutated residues in 9 orthologous (vertebrate) GALC proteins. The computational analysis, carried out at http://www.ensembl.org/, revealed that all 18 missense mutations occurred at amino acid residues which were evolutionarily conserved in chicken as well as in various mammals. Further, eleven of the residues involved (including those harbouring the novel p.P318R, p.G323R and p.Y490N mutations) were invariant even when zebrafish was considered (Supp. Table S4).

When a similar analysis was performed on the missense polymorphisms (Supp. Table S5), one of the two SNPs predicted by *MutPred* to be potentially deleterious (p.A21P) was found to occur in a residue which was conserved in 5 vertebrates and hence could therefore be of functional significance. However, residue 641, which harbours the other potentially functional p.T641A (T625A) SNP, was not evolutionarily conserved. The relationship between *MutPred* values and residue evolutionary conservation therefore appears to be rather more tenuous in the case of the missense polymorphisms than it is for the missense mutations. Thus, the residue harbouring the D248N (D232N) SNP was conserved in 7 vertebrates including the three-spined stickleback, *Gasterosteus aculeatus*, even although its *MutPred* score was 0.52 (whilst residue 562, harbouring the p.I562T SNP allele (*MutPred* score 0.53), was not evolutionarily conserved). Intriguingly, this latter SNP was present in all three patients with an unknown *GALC* allele but, since it is also present in 14 other patients, it is not possible to draw any firm conclusions as to its possible functional significance.

### Consequences of *GALC* mutations for mRNA processing

Reverse transcript-polymerase chain reaction (RT-PCR) analysis was used, wherever possible, to investigate frameshift, nonsense and splicing mutations to assess their effect on *GALC* mRNA processing.

#### Frameshifts and stop codons

A total of 6 microdeletions, 2 insertion/deletions (indels) and 2 microduplications were identified during the course of this study. Most were explicable in terms of slipped mispairing between direct repeats or through the deletion of a single base within a mononucleotide tract (see Supp. Table S6). Nine of these mutant alleles (seven previously unreported) resulted in frameshifts and were therefore analysed by RT-PCR. Although the analyses revealed RT-PCR products of a size equivalent to that expected of the wild-type in the case of p.E136EfsX35, p.Y174LfsX3, p.H391IfsX65 and p.L469YfsX22, no product was present when the results were evaluated for p.A21RfsX51, p.G102GfsX5 and p.G609GfsX6 (data not shown). One explanation for these findings could be that the latter three premature stop codons elicited nonsense-mediated mRNA decay (NMD) and hence the corresponding abnormal transcripts would have undergone degradation [Nicholson et al, 2010].

**Figure 2:**
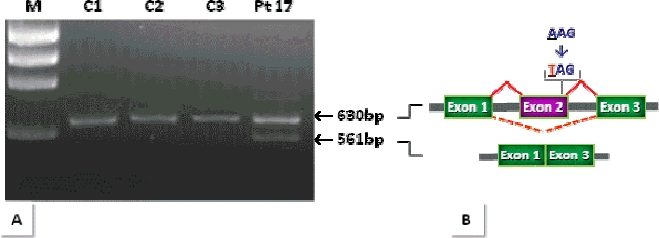
RT-PCR on patient (Pt) #17 [p.K88X]+[p.Y490N]. **A:** RT-PCR analysis performed on the RNA sample using primers encompassing exons 1-6 (Supp. Table S1), revealing the presence of an abnormally shorter *GALC* transcript (561-bp) in addition to the normally-sized product (630-bp). **B:** The graphical representation of the result, confirmed by sequencing the two cloned products. The analysis demonstrated normal splicing (unbroken red line) of the 630-bp fragment and abnormal splicing (dotted red line) of the 561-bp product resulting in a 69-bp in-frame deletion corresponding to the entire exon 2 of *GALC* in the shorter product. The AAG>TAG transversion, affecting the last codon of exon 2, is given. Note that only exons 1, 2 and 3 are graphically shown. M, marker = ϕX 174 DNA *Hae*III-digested; C1, C2, C3 = control samples.

RT-PCR analyses were also performed to analyse transcripts bearing the three novel stop codons (p.R69X, p.K88X and p.R127X). Whereas the transcript harbouring p.R127X appears to be unstable (since the *GALC* mutation *in trans* and the associated SNPs were invariably found in apparent homozygosity in patient #16), the transcript harbouring p.R69X was found to be expressed. The situation pertaining with the p.K88X mutation (occurring in *trans* to p.Y490N in patient #17) was however found to be more complex in that it also impacts on splicing. An RT-PCR-fragment spanning exons 1-6 of the *GALC* gene yielded two products in this patient, one of the expected size (630 bp), probably carrying the p.Y490N missense mutation, and one smaller (561 bp). Both fragments were cloned and sequenced; although the 603 bp fragment did not exhibit any alteration within the region analysed, the smaller 561 bp fragment was found to contain a 69 bp in-frame deletion corresponding to the entire length of exon 2 (Figure 2). It would appear that the c.262A>T transversion, occurring in the first base of the last codon (AAG) in this exon, affects one of the variant donor splice sites of exon 2, introducing a premature stop codon at its last residue (K88X). Although it is likely that the mutation directly abrogated the canonical exon 2 donor splice site, it is also possible that the splicing machinery acted so as to restore the open reading frame simply by removing exon 2 in its entirety [Nissim-Rafinia and Kerem, 2002].

#### Splicing mutation

**Figure 3:**
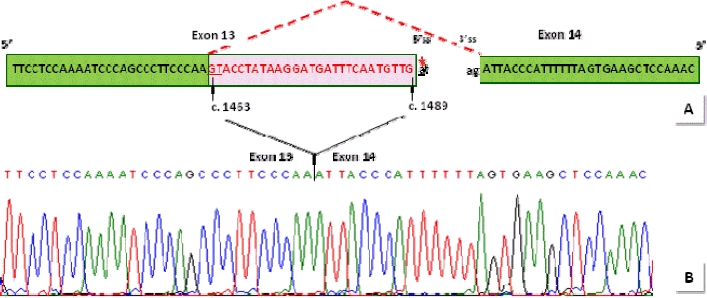
Schematic representation of the abnormal *GALC* splice variant in patient #20. In the top half of the diagram (A), red dotted lines represent the aberrant splicing event which results from the c.1489+G>A mutation within the invariant gt dinucleotide at the 5′ splice site (ss) (marked by a red arrow). As a consequence of the aberrant splicing event, the junction between nucleotides c.1462 (exon 13) and c.1490 (exon 14) occurs at a novel donor splice site, almost certainly mediated by the use of the exonic (underlined) GT dinucleotide at c.1463-1464. In the bottom half of the diagram (B), a sequence analysis chromatograph of an RT-PCR product from the patient shows the aberrantly non-canonical exon 13-14 junction at c.1462-c.1490 instead of c.1489-c.1490, consequent to the loss of 27 nucleotides (red type). Green boxes denote the portion of the genomic sequence of exons 13 and 14 that is presented in the chromatograph (B); the pink box denotes the skipped 27 nucleotides of exon 13. The black dotted line represents intron 13.

To confirm the pathological authenticity of the intronic mutation c. 1489+1G>A, RT-PCR analysis was performed on the mRNA of patient #20. In this patient, the mutation occurred in *trans* to the rare microdeletion c.1901delT (1853delT) that was predicted to introduce a premature stop codon at residue 634 (p.L634X) (L618X) (Table 1). The c.1489+1G>A transition, occurring within the invariant GT dinucleotide of the intron 13 donor splice-site, was expected to lead to the skipping of exon 13 by abolishing the canonical splice site. However, contrary to expectation, RT-PCR analysis of *GALC* RNA from the patient (using a primer set spanning exons 10-17, as reported in Supp. Table S1) revealed the presence of an abnormally short transcript in addition to the normally-sized 1089 bp products (data not shown), both of which were cloned and sequenced. Although sequence analysis of the two RT-PCR products confirmed the presence of the microdeletion c.1901delT in the apparently normally-sized cDNA product, only partial skipping of exon 13 was evident, with the shorter transcript lacking the last 27 nucleotides (c.1463_1489) (Figure 3). The junction between nucleotides c.1462 (exon 13) and c.1490 (exon 14) at the new donor splice-site (probably mediated by the use of the cryptic exonic GT dinucleotide at c. 1463-1464), gave rise to a serine to asparagine substitution at residue 495 followed by a frameshift that is predicted to lead to the premature termination of translation (p.S488NfsX200).

### Insights into the genotype-phenotype relationship in Krabbe disease

Table 1 summarizes the clinical phenotypes of the 30 Krabbe disease patients, most of whom presented with the clinically severe infantile form. Owing to the highly heterogeneous *GALC* mutational profile, it is difficult to discern any general trends in terms of a genotype-phenotype relationship. In addition to the mutational heterogeneity, the highly variable polymorphic background manifested by each patient (Supp. Table S3) could also play a role in modulating the genotype-phenotype relationship.

Consistent with previous studies, the common 30kb deletion (c. 1161+6532_polyA+9kbdel) was invariably found in *cis* to c.550T (502T), and frequently in patients with the infantile form of the disease (but sometimes also with the juvenile form, depending on the precise combination with the other mutant alleles). Our series of patients also provided support for previous speculation that the p.G286D (G270D) mutation might be associated specifically with the juvenile/mild forms of Krabbe disease [Furuya et al., 1997; De Gasperi et al., 1999]. Indeed, the juvenile form of the disease in patient #26 (Table 1), carrying the large 30kb deletion (c.1161+6532_polyA+9kbdel) on one *GALC* allele, may have been consequent to the contribution of the putatively milder p.G286D (G270D) lesion on the second allele. Further evidence to support the less deleterious nature of this missense mutation was provided by the adult form of the disease in patient #30 (Table 1) in whom p.G286D (G270D) occurred in *trans* to the novel (and probably highly deleterious) p.P318R mutation. The replacement of a cyclic uncharged proline (P) with a basic charged arginine (R) in position 318 was predicted by *MutPred* to impact upon protein structure both in terms of solvent accessibility (P = 0.0179) and secondary structure (loop → helix; P = 0.0259) (Table 2). Another *in silico* hypothesis was available for the novel missense mutation p.Y490N: the non-conservative substitution of tyrosine (Y) by asparagine (N) is predicted to lead to the loss of a phosphorylation site at residue 490 (P = 0.0245), consistent with the severe infantile form of the patient #17 (Table 1). The second allele in this patient (#17) is the nonsense mutation (p.K88X), discussed above, that predicts premature termination of translation at codon 88.

In accordance with *in vitro* expression studies which indicated that no GALC enzymatic activity was evident in association with p.G553R (G537R) [De Gasperi et al., 1999], *MutPred* analysis postulated a change in the alpha-helical secondary structure as consequence of the replacement of a glycine with an arginine at residue 553. Consistent with this prediction, we found this mutation in association with the severe infantile form of the disease either in homozygosity (Table 1, pts #3, #14, #19) or compound heterozygosity (Table 1, pts #4, #11, #16). Only once was p.G553R (G537R) found in association with a late-onset form of the disease (Table 1, pt #29); in this case, the less severe phenotype was probably due to the contribution of p.R79H (R63H) on the second allele, confirming an already reported association with the late-onset form of the disease [De Gasperi et al., 1999].

The third most frequent mutation, p.N295T (N279T), was found in two infantile onset patients (#8 and #15) in *trans* to p.E130K (E114K) and the common large 30kb deletion, respectively. The same p.N295T (N279T) mutation was also found in compound heterozygosity in a patient (#28) with the juvenile form of the disease, in association with the novel frameshift mutation, p.G609GfsX6. These observations are not only suggestive of a highly detrimental effect for the p.E130K (E114K) mutation (first reported by Lissens et al., 2007) but also allow us to postulate a less severe phenotype in association with the novel frameshift mutation, p.G609GfsX6.

Finally, examination of our patient series revealed a possible association with the juvenile form of the disease for the novel missense mutation p.I384T, found in patient #27 (Table 1) in *trans* to the novel nonsense p.R69X mutation (expected to introduce a premature termination codon; see above).

To conclude, despite the highly heterogeneous mutational and polymorphic profiles of the *GALC* gene, studies on our large series of Krabbe disease patients have provided support for various (previously somewhat tentative) genotype-phenotype correlations that were based on relatively small numbers of patients with extremely rare mutations. In addition, the present data suggest that the *GALC* mutational spectrum underlying Krabbe disease in the Italian population is somewhat different from that reported in other patient cohorts with European ancestry. This appears to be due, at least in part, to a prevalent *GALC* missense substitution p.G553R (G537R) whose high frequency appears to be due to a founder mutation. Other known examples of *GALC* founder mutations include the large common deletion (c.1161+6532_polyA+9kbdel) thought to have originated in Sweden [Wenger et al., 1997], the two mutations c.1796>G and c.1630G>A (1748T>G and 1582G>A) present in the Druze and Moslem Arab populations [Rafi et al., 1996] and c.169G>A (121G>A), which has been reported as being responsible for the high incidence of Krabbe disease in a restricted geographical area of southern Italy (Catania, Sicily) [Lissens et al., 2007].

## Figures and Tables

**Supp. Table S1:** List of primers used for polymerase chain reaction (PCR) amplification of *GALC* gene exons and exon–intron boundaries

Exons amplified	5′ sequence flanking exon (bp)	Forward (5′>3′)*	Reverse (5′>3′)*	3′ sequence flanking exon (bp)	Annealing Temperature (°C)	PCR product size (bp)
**Genomic DNA Amplification**§						
1	86 #	GTCAGCATCAGCGGCCTCCT	ACTGGCACCCTAGGGGAAT	134	62	415

2-3	169	CAATGATTGGCACAGAAAGG	TCACAGTCCATATGCTGAGGT	70	58	619**

4	72	GGTGGGGAGTGAGATGGTC	CAAGGGCAAAGAAAGGATCA	172	58	359

5	64	TATTTTCAATAGCGCCAGCA	GCAAAGGGGAGCAATTAAAG	131	50	381

6	71	TCGTAACGATAATCTGCTTTCTG	GGTATTTCCAACACAAATTTC	101	60	211

7	77	ATCTTGGTCATAAATTCAACAGC	GAGAATGTAATCAAATGGGGAGA	98	60	305

8	182	TGCTGAGACAAAAGGGCATA	GATGACGCTAACAAGGCAAA	133	58	471

9	131	TTGGGTGATCCTTTTATTGTCA	CACTGGCAAATCTTGCTTAAAA	140	58	397

10	106	GTTTGGATGAATCAGACTCAAAT	TGGCATCTGTCTGTATGCTTATG	116	58	350

11	97	AAATTTCTGTTAATCTTGGGCATT	GAACTACTGGCCTGTGACAGAA	73	60	259

12	81	TCTTGCTGGTACTGATTTTGGA	TGACATTTCTGTGCCCTTTT	71	60	239

13	63	TTCTGTCCACATGAGATGAGC	CATCATGCACCCAGTTTGAC	86	60	300

14	60	AGCAAGGAGAGCTTCTGAAGGA	AGGTTCTTGAAATAGGAGGACCA	107	60	347

15	154	TCTTGAAGCCCATCTCTGCT	TGCTTACATCCCTTCCCAAT	108	60	456

16	86	CCACTCAAGAACCCCACTGA	GTCACACTTTCCCCCTCCTA	94	60	256

17	140	GGAATTGTGTTTTGCTGTGG	ACCAGTTTTCCCCTTGGAAT	200 ##	60	487

**cDNA Amplification**°						

1-6		GAGTCATGTGACCCACACAA	TTGGCATTATATGACCTCTCAT		62	630

1-10		GAGTCATGTGACCCACACAA	TAAGCCATCAGTCAGAGCTACG		65	1150

10-17		AGTTTACTCAACCTGGCTGG	AACAAGAATTGGCTCTGAACC		59	1089

§ *GALC* gene: GenBank-EMBLaccession no. NC_000014.8

° *GALC* cDNA GenBank-EMBL accession no. NM_000153.2

* the primersare located 5′ (upstream) and 3′ (downstream) to the intronic sequences flanking each exon

** in this case, the size also includes intron 2; part of genomic region at 5′ UTR(#)and 3′ UTR(##)

**Supp. Table S2:** Oligonucleotide primers used for PCR amplification of the novel missense mutations and polymorphisms

Location		Forward (5′>3′)#	Reverse (5′>3′)#	Annealing Temperatur e (°C)	PCR product size (bp)	Restriction enzyme
**Mutation**§						

Ex. 9	c.953C>G	TTGGGTGATCCTTTTATTGTCA	CAACCCGCATCTCCCATGA*	58	195	Mnl I

Ex. 9	c.967G>A	AGTTGCCTTATGGGAGCTGC*	CACTGGCAAATCTTGCTTAAAA	60	229	Pst I

Ex. 10	c.1151T>C	GTTTGGATGAATCAGACTCAAAT	ACAAAAGTTTACCATGGTTCCA*	56	246	Bsr I

Ex. 13	c.1468T>A	TTCTGTCCACATGAGATGAGC	CCAACATTGAAATCCACCTTAT*	59	215	Bstx I

**Polymorphism**§						

UTR 5′	c.42GC	GCTTCCTGGCAACGCCGATC*	ACTCATGGCCCTCTTCCTTT	62	309	Taq I

Ex. 1	c.61GC	GTCAGCATCAGCGGCCTCCT	ACTCATGGCCCTCTTCCTTT	62	415	Sma I

Ex. 1	c.75CA	GTCAGCATCAGCGGCCTCCT	ACTCATGGCCCTCTTCCTTT	62	415	Hga I

Intr. 1	c.195+34GT	GTCAGCATCAGCGGCCTCCT	ACTCATGGCCCTCTTCCTTT	62	415	Bbv I

Intr. 7	c.752+56TC	ATCTTGGTCATAAATTCAACAGC	GAGAATGTAATCAAATGGGGAGA	60	305	Taq I

Legend: Ex=exon, Intr=intron;

§ *GALC* gene GenBank-EM BL accession no. NM_000153.2 and no. NC_000014.8

* primer modified so asto introduce a new restriction enzyme cleavage site according to Schwartz et al. (1991)

**Supp. Table S3:** Characteristics of putatively neutral SNPs identified in the *GALC* gene

					MutPred analysis ♦		
						
Position	dsSNP rs no.	Traditional numbering nucleotide (amino acid) substitution	Site of nucleotide substitution* (amino acid change)**	Minor allele frequency§	Probability of deleterious mutation	Confident in-silico hypotheses	Polymorphic background in the Patients #
**UTR5′**			**c.42GC (p.A14A)**	**0.22**	-		10,11,16

**Ex. 1**			**c.61GC (p.A21P)**	**0.15**	**0.62**	**None**	10,16

**Ex. 1**			**c.75CA (p.G25G)**	**0.11**	-		10,11,16

**Intr. 1**			**c.195+34GT**	**0.17**	-		10,16

**Intr. 2**	rs2245387	216+108GA	c.264+108GA	0.48	-		5,6,7,8,15,18,21,22,24,25,27,28

**Intr. 3**			**c.328+19TA**	NA	-		10

**Ex. 4**	rs11552556	282CT (D94D)	c.330CT (p.D110D)	NA	-		23,30

**Ex. 5**	rs1805078	502CT (R168C)	c.550CT (p.R184C)	0.47	0.38	None	1,7,9,10,12,15,18,23,24,26

**Ex. 7**	rs34362748	694GA (D232N)	c.742GA (p.D248N)	0.09	0.52	None	10,16

**Intr. 7**			**c.752+56TC**	**0.13**	-		16

**Ex. 9**	rs12888666	936GA (Q312Q)	c.984GA (p.Q328Q)	0.48	-		5,6,7,8,15,18,21,22,24,25,27

**Ex. 10**	rs74073730	1024CT (L342L)	c.1072CT (p.L358L)	NA	-		3,4,11,14,16,19,29

**Intr. 10**	rs17687109	1113+38TC	c.1161+38TC	0.08	-		10

**Intr. 10**	rs11300320	1114-4delT	c.1162-4delT	NA	-		1,4,7,8,13,15,16,17,18,21,24,30

**Intr. 12**	rs74076317	1290+23TC	c.1338+23TC	NA	-		3,4,11,14,16,19,29

**Ex. 13**	rs398076	1302CT (S434S)	c.1350CT (p.S450S)	0.44	-		1,2,6,11,12,13,17,18,21,23,24,25,26,27,28,29,30

**Ex. 14**	rs367327	1572GA (T524T)	c.1620GA (p.T540T)	0.15	-		1,2,5,6,7,8,9,10,11,13,15,16,17,18,19,20,21,22,23,24,25,28,29

**Ex. 14**	rs9672064	1584TC (D528D)	c.1632TC (p.D544D)	NA	-		19

**Intr. 14**	rs366615	1622+60CT	c.1670+60CT	0.48	-		1,4,9,11,19,21,22,24,27,29

**Intr. 14**	rs12432149	1623-15CT	c.1671-15CT	0.48	-		5,7,8,10,15,16,17,20,21,22,24,25,27,28,29,30

**Ex. 15**	rs398607	1637TC (I546T)	c.1685TC (p.I562T)	0.47	0.53	None	1,2,4,6,9,12,13,17,21,22,23,24,25,27,28,29,30

**Ex. 15**	rs421466	1650AT (V550V)	c.1698AT (p.V566V)	0.17	-		1,2,4,5,6,7,8,9,10,11,12,13,15,16,17,18,20,21,22,23,25,28,29,30

**Intr. 15**	rs448805	1786+5CG	c.1834+5CG	0.19	-		1,4,8,11,16,28,29,30

**Ex. 17**	rs421262	1873AG (T625A)	c.1921AG (p.T641A)	0.17	0.58	None	1,2,4,5,6,7,8,9,10,11,12,13,15,17,18,20,21,22,24,25,28,29,30

Legend: dbSNP, the Single Nucleotide Polymorphism database, is available at http://www.ncbi.nlm.nih.gov/projects/SNP/

* GenBank-EMBL accession no. NM 000153.2

** GenBank-EMBL accession no. NP 000144.2

§ the minor allele frequencies were available at dbSNP or calculated from 200 Italian control alleles (novel polymorphisms, in bold)

NA: not available

# patient number according to Table 1.

♦ Li et al., (2009) and Mort et al., (2010).

## Supplementary material

Supp. Table S4. Evolutionary comparison of the protein sequences flanking the missense mutations identified in the human GALC protein with their orthologous counterparts in nine eukaryotes

Supp. Table S5. Evolutionary comparison of the protein sequences flanking five missense SNPs identified in the human GALC protein with their orthologous counterparts in nine eukaryote

Supp. Table S6. Different types of repeats (underlined and highlighted in yellow) that could have mediated micro-deletions, micro-insertion/deletions (indel) and micro-duplications in the *GALC* gene.

Mutated nucleotides are shown in bold upper-case red letters.
