# Supplementary figures and images for: Identification and Characterization of 15 Novel GALC Gene Mutations Causing Krabbe Disease
Source: Hum Mutat. 2010 Dec;31(12):E1894–915. doi: 10.1002/humu.21367 (PMC3052420; doi:10.1002/humu.21367)

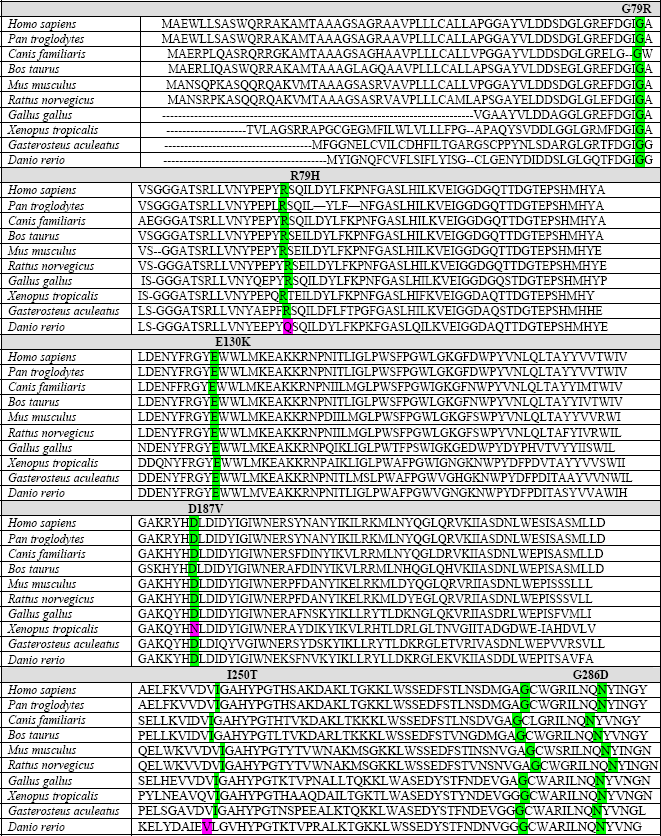

Supplement: Supplementary file 1 [file humu0031-E1894-SD1.gif]

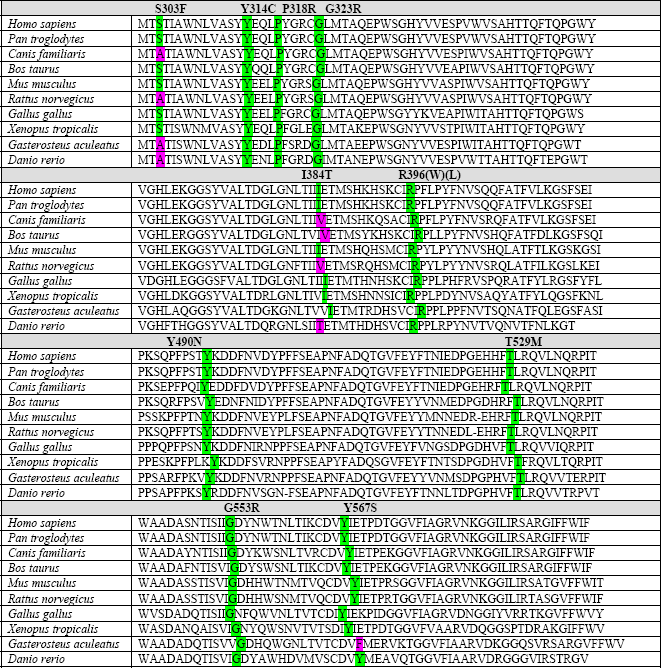

Supplement: Supplementary file 2 [file humu0031-E1894-SD2.gif]

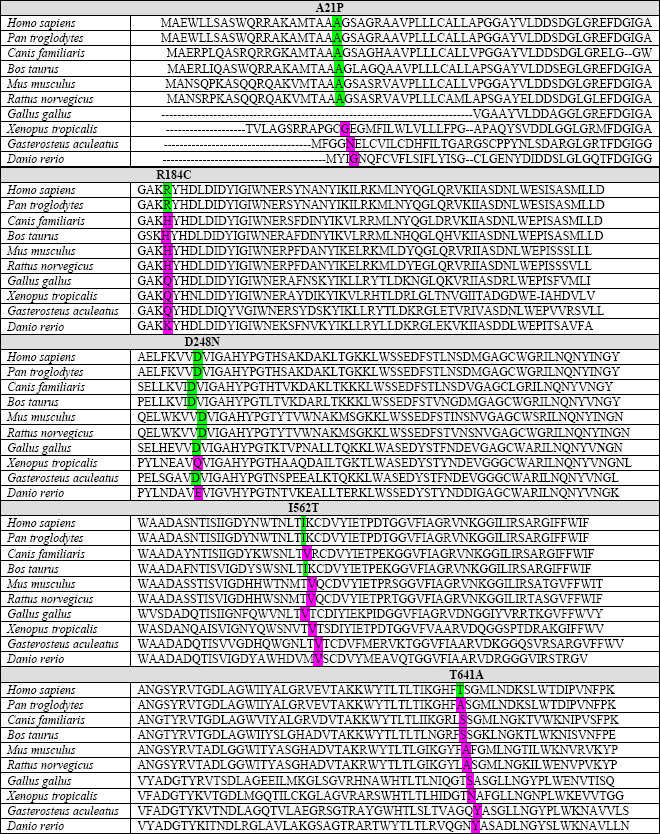

Supplement: Supplementary file 3 [file humu0031-E1894-SD3.gif]

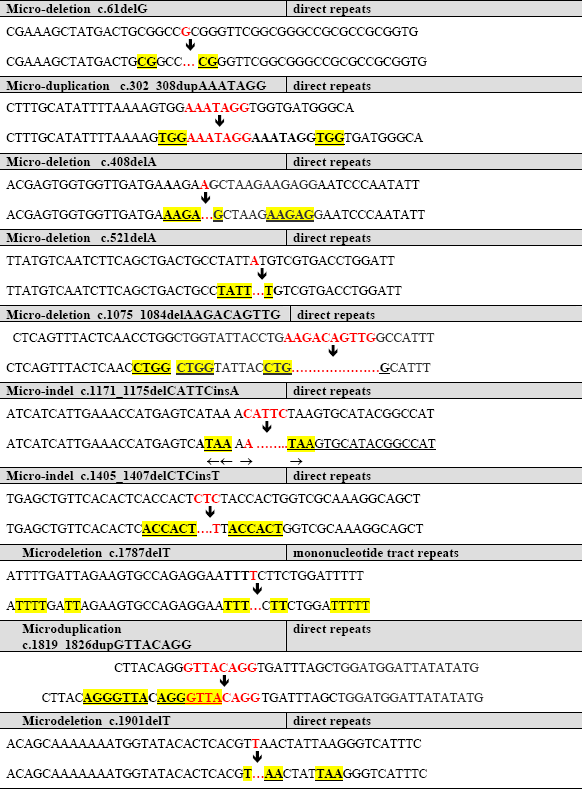

Supplement: Supplementary file 4 [file humu0031-E1894-SD4.gif]
